# A Highly Sensitive Immunoassay for Determination of Immune Response to SARS-CoV-2 in Capillary Blood Samples

**DOI:** 10.3390/biomedicines10112897

**Published:** 2022-11-11

**Authors:** Belén G. Sánchez, Alicia Bort, José María Mora-Rodríguez, Alba Díaz-Yuste, José Manuel Gasalla, Manuel Sánchez-Chapado, Alba Sebastián-Martín, Inés Díaz-Laviada

**Affiliations:** 1 Biochemistry and Molecular Biology Unit, Department of Systems Biology, School of Medicine and Health Sciences, University of Alcalá, 28871 Alcalá de Henares, Madrid, Spain; 2 Department of Comparative Medicine, School of Medicine, Yale University, New Haven, CT 06519, USA; 3 Clinical Biochemistry Service, Príncipe de Asturias Hospital, 28805 Alcalá de Henares, Madrid, Spain; 4 Department of Urology, Príncipe de Asturias Hospital, 28805 Alcalá de Henares, Madrid, Spain; 5 Department of Surgery, Medical and Social Sciences, School of Medicine and Health Sciences, University of Alcalá, 28871 Alcalá de Henares, Madrid, Spain; 6 Chemical Research Institute “Andrés M. del Río” (IQAR), Alcalá University, 28871 Alcalá de Henares, Madrid, Spain

**Keywords:** COVID-19, SARS-CoV-2, ELISA, IgG, serology, point-of-care, capillary blood

## Abstract

Throughout the pandemic, serological assays have been revealed as crucial for detecting previous exposures to the virus and determining the timing of antibody maintenance after vaccination or natural infection. This study aimed to develop an optimized enzyme-linked immunosorbent assay (ELISA)-based serology, which could be used in case of reagent shortages, such as that occurred in the beginning of this health emergency. As a result, we present a high-sensitive immunoassay for the determination of IgG levels in venous serum samples, using 2 μg/mL antigen (receptor-binding domain of the spike protein S1) for coating the plate and utilizing human samples at a dilution 1:1000. This method showed non-inferiority features versus a commercial kit, is less expensive, and has a higher spectrophotometric range that allows for a better quantification of the antibody titers. The optical density values before and after heating venous serum samples at 56 °C during 30 min was quite similar, showing that heat inactivation can be used to reduce the biohazardous risks while handling samples. Furthermore, we show that finger-stick capillary blood samples can also serve as a suitable source for IgG detection, bypassing the need for serum isolation and being suitable for point-of-care application (Pearson’s coefficient correlation with capillary serum was 0.95, being statistically significant).

## 1. Introduction

The coronavirus disease 2019 (COVID-19), caused by severe acute respiratory syndrome coronavirus 2 (SARS-CoV-2), is still considered a public health emergency of international concern. Since the onset of the pandemic, there has been an urgent need for rapid and reliable methods for detecting SARS-CoV-2. Thus, nasopharyngeal, oropharyngeal, and saliva samples have been used for testing the presence of SARS-CoV-2 nucleic acids by the polymerase chain reaction (PCR) technique [1,2,3]. Shortly after, more intrusive and uncomfortable procedures of sample collection, such as anal swabs, were utilized in China or Indonesia [4,5]. Subsequently, while time, outbreaks, and vaccination progressed, other features of the clinical course became relevant for the understanding of the pandemic, such as the immune system response.

In this context, serological assays emerged as a powerful tool in the management of this global health issue. Firstly, as an indirect measure of the virus, given that antibodies constitute the host’s response to infection and, in that sense, serology allows determining previous exposures to the virus and the extent of the viral spread by performing epidemiological seroconversion studies [6,7,8]. Nonetheless, during SARS-CoV-2 infection, antibody responses are variable and generate controversy in terms of immunity and persistence [9]. Secondly, it is crucial to determine the timing for antibody maintenance after vaccination or even a natural infection [10,11]. This fact acquired a special relevance given that SARS-CoV-2 vaccines, which have already entered phase III clinical trials, arose as a critical solution [12]. The measurement of specific COVID-19 immunoglobulins (both IgG and IgM) can be used to verify the effectiveness of the different vaccines in the population, although antibody absence does not necessarily indicate a lack of immunization, given that T-cell immune responses have been reported after vaccination also in the absence of seroconversion in patients with lymphoid malignancies [13]. Within both, IgG seems more appropriate for a general screening given that it is more prominent in blood and remains high for a longer time than IgM [14]. Venous blood was standardized to measure levels of anti-SARS-CoV-2 antibodies, along with studies that try to correlate viral positivity, seroconversion, and disease severity in COVID-19 [14].

A key feature of robust serology tests is their high analytical and diagnostic sensitivity, which is important to detect low antibody titers. In addition, serology assays need to be cost-effective, high-throughput, scalable, and easy to implement in order to be useful for public worldwide emergencies. Enzyme-linked immunosorbent assay (ELISA)-based serological tests are usually commercialized as kits that contain all reagents necessary to perform the assay. However, despite their precision and sensitivity, those kits are expensive and hardly affordable during economic crises or disfavoured economical situations in a country. Furthermore, in emergency contexts, such as thatoccurred in the early COVID-19 pandemic, there may be shortages of reagents, due to their enormous demand, restrictions of transports, and limitations of delivery, hampering their acquisition. This happened all around the world [15] but specially affected developing countries and areas with less infrastructure adaptation and capacity planning [16]. Therefore, alternatives were required to surmount supply chain limitations [17,18], as well as the shortage of healthcare professionals [19]. Despite huge efforts, alternatives are still required to replace commercial kits, lowering their cost if necessary, and allowing the scientific community to support high-throughput screenings.

Point-of-care antibody measurement may be important in some situations, for example, in outpatient settings, in small hospitals, or to reduce time delays when results are needed urgently in the emergency department [20]. One of the often-found difficulties of point-of-care tests is sample collection, which, in the case of serological assays via venepuncture, must be performed by health professionals to avoid infection. The handling of these samples and the use of a needle makes that the collection requires a high degree of expertise due to the associated biohazardous risks, together with the need of a laboratory with a certain biological safety level (BSL) and specific protocols for the disposal of bio-waste. Moreover, once blood is collected, it should be centrifuged to separate the serum from blood cells. Although this is simple when a centrifuge is available, this process can complicate the analysis at the point-of-care. In addition, large-scale and iterative serological testing by venous blood draw in older persons can be challenging [21].

Interestingly, capillary blood sampling is a low-invasive procedure requiring smaller amounts of blood volume and can be quickly and easily performed [22] and therefore is a promising alternative to venepuncture. Finger-stick sampling is also becoming more common in research and clinical settings. It can be self-managed by people at home and used even for remote collection without requiring professional assistance [21]. Moreover, it avoids exposure risks between clinical staff and patients. This technique allows point-of-care testing and is beneficial to reduce the risks involved in the preanalytical phase. However, not every analyte can be measured in serum or plasma from venous and capillary samples. Therefore, it is essential to optimize assays and guarantee an accurate test without affecting the validity of the tested results.

We aimed to develop an ELISA-based serology protocol to measure anti-SARS-CoV-2 IgGs, utilizing readily available reagents and instruments so that they could be applied easily in situations of reagent supply restrictions. This procedure was optimized using venous serum human samples, obtaining a method that can also be employed in finger-stick capillary blood as well as in capillary serum. Results were compared with those obtained in a commercially available ELISA kit, along with the data of a PCR test. We showed that capillary blood is suitable for serological analysis to detect IgG against the receptor-binding domain (RBD) of the spike protein S1 of SARS-CoV-2.

## 2. Materials and Methods

### 2.1. Patients and Samples

A total of 123 venous serum samples were collected during a span of 30 days (from 1 to 30 June 2020) by health care workers from the Príncipe de Asturias University Hospital (Alcalá de Henares, Spain). These samples were traditionally collected through patient venepuncture in evacuated tubes containing lithium heparin anticoagulant as well as gel for plasma separation. Samples were then centrifuged and stored at −20 °C until the experimental phase. Of them, 98 were from patients hospitalized in said Hospital because of COVID-19-related symptoms, such as fever, cough, severe respiratory distress, bifocal pneumonia determined by chest X-ray, and SpO2 < 93% in room air. In addition to the clinical diagnostic, these patients had a positive result in a reverse-transcription qualitative real-time polymerase chain reaction (rRT-PCR) test when assaying a nasopharyngeal swab. The rRT-PCR was performed with the commercial kit Allplex^TM^ SARS-CoV-2 Assay (Seegene Inc, Seul, South Korea), designed for the detection of 3 target genes for SARS-CoV-2 (N, S, and RdRP genes), along with an internal control, using a CFX96 Touch Real-Time PCR Detection System (Bio-Rad Laboratories Inc, Hercules, CA, USA). The accuracy of the method depends on specific SARS-CoV-2 lineages and variants to be detected [23,24,25,26,27].

Inclusion criteria include: age >18 years (adult or older adult), both sexes were eligible, a clinical diagnosis of COVID-19, and a positive rRT-PCR test. Exclusion criteria include: hospitalization for non-COVID-19-related causes and COVID-19 diagnosis not checked by a positive rRT-PCR test. The remaining 25 patients constitute the control group as they went to the hospital for other reasons and had a negative rRT-PCR result for SARS-CoV-2. Demographic data and clinical features were available and collected according to the patient record system, although no conclusions were extracted considering their sex, age, or others, since samples were only utilized for optimizing a method or protocol. Briefly, in the COVID-19 group, 65% were men and 35% women, with a median age (interquartile range, IQR) of 56 (53–72) and 58 (57–69), respectively; while the control group was made up of men with a median age of 69 (66–71) years old. Data collection occurred during first-time examination at admission (within 24 h after admission).

The study was approved by the Ethics Committees of the Príncipe de Asturias Hospital and Alcalá University (LIB21-2020 and CEI/HU/202/37) and conforms to the principles outlined in the Declaration of Helsinki. Consent statement management is detailed at the end of the paper.

### 2.2. Capillary Blood Collection

Capillary blood was collected in capillary tubes coated with tripotassium EDTA (K3EDTA) (Greiner Bio-one, Madrid, Spain). For this purpose, forty volunteers were chosen to whom the self-sampling procedure was explained by the investigator. Participants were advised to place their non-dominant hand in warm water for 1–3 min, subsequently drying it. After cleaning one finger with a solution of alcohol 70%, it was punctured with a 1.25 mm contact-activated Minicollect^®^ 28G lancet (Greiner Bio-one, Madrid, Spain). Blood flow was encouraged by massaging the finger in the direction of the puncture site until 50 µL of blood had been collected in the K3EDTA tube. Capillary serum was then obtained by centrifuging for 10 min at 1500× *g*.

### 2.3. Commercially Available ELISA Serologic Assay

The human IgG anti-S1 protein (RBD region) of SARS-CoV-2 was determined in patient serum samples using an ELISA Detection Kit (GenScript USA, Inc., Piscataway Township, NY, USA) (Ref. L00831). The manufacturer’s instructions were followed and then serum samples were diluted to 1:100.

### 2.4. Optimized ELISA-Based Serology Assay

**Figure 1:**
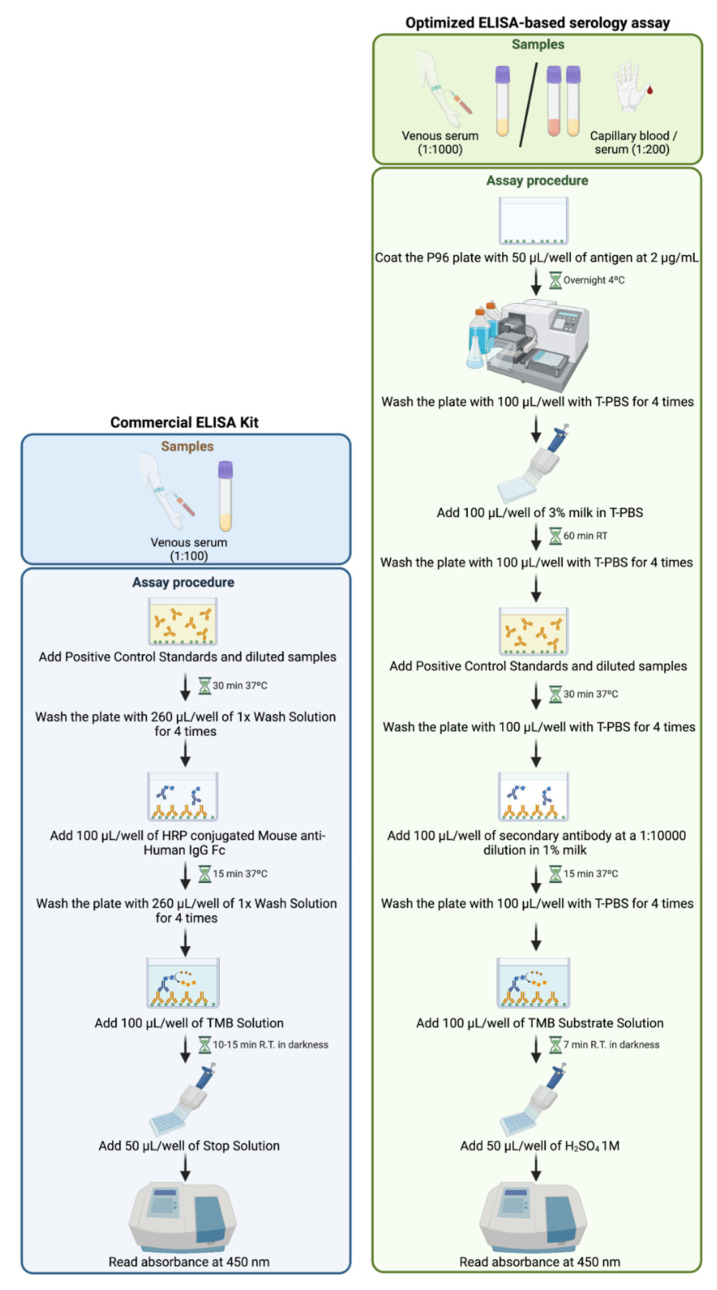
**Schematic representation of ELISA protocol, comparing both methods.** On the left, commercially available ELISA Detection Kit (GenScript USA Inc., Piscataway, NJ, USA). On the right, protocol for the optimized ELISA-based serology assay described in this paper.

The protocol is represented in Figure 1 (in comparison to the commercial ELISA kit) and proceeds as follows.

#### 2.4.1. Materials

The antigen, the RBD of the SARS-CoV-2 Spike protein S1 (Ref. Z03501-100), and the IgG positive control (Ref. L00831-10) were obtained from GenScript USA, Inc. (Piscataway Township, NY, USA). Horseradish peroxidase (HRP)-conjugated mouse anti-human IgG Fc (secondary antibody) was from Abcam (Cambridge, UK) (Ref. ab99759) and TMB (3,3′,5,5′-Tetramethylbenzidine). Substrate Solution was from Thermo Fisher Scientific (Rockford, IL, USA) (Ref. N301). P96 plates (Ref. 442404) and plate caps (Ref. AB0674) were obtained from Thermo Fisher Scientific (Rockford, IL, USA).

#### 2.4.2. Reagents

- Wash reagent: Tween 0.1% in phosphate-buffered saline (T-PBS).
- Standard and antigen dilution reagent: carbonic acid (H_2_CO_3_) buffer 0.2 M at pH 9.4.
- Sample diluent reagent: T-PBS.
- Blocking reagent: milk 3% in T-PBS.
- Stop solution: sulfuric acid (H_2_SO_4_) 1M.

#### 2.4.3. Sample Preparation

1. Venous serum: samples are diluted to 1:1000 in T-PBS.
2. Capillary blood or capillary serum: samples are diluted to 1:200 in T-PBS.

#### 2.4.4. Assay Procedure

1. Coat the P96 plate with 50 µL/well of 2 µg/mL recombinant RBD of the SARS-CoV-2 S1 spike protein in dilution reagent.
2. Cover the P96 plate and incubate at 4 °C overnight.
3. Remove the lid and wash the plate with 100 µL/well of T-PBS 4 times.
4. Tap the plate on a paper towel to remove any remaining buffer from washes.
5. Add 100 µL of 3% milk in T-PBS.
6. Cover the plate and incubate for 1 h at room temperature.
7. Remove the lid and wash the plate with 100 µL/well of T-PBS 4 times.
8. Tap the plate on a paper towel to remove any remaining buffer from washes.
9. Add 100 µL of Positive Control Standards or the diluted human samples per well, cover the plate, and incubate for 30 min at 37 °C.
10. Remove the lid and wash the plate with 100 µL of T-PBS 4 times.
11. Tap the plate on a paper towel to remove any remaining buffer from washes.
12. Add 100 µL of secondary antibody at a 1:10,000 dilution in 1% milk and incubate for 15 min at 37 °C.
13. Remove the lid and wash the plate with 100 µL of T-PBS 4 times.
14. Tap the plate on a paper towel to remove any remaining buffer from washes.
15. Add 100 µL of TMB Substrate Solution per well and incubate the plate in the dark for 7 min at room temperature.
16. Add 50 µL per well of H_2_SO_4_ 1M.
17. Read absorbance immediately on a spectrophotometer at 450 nm.

Note: To start the ELISA protocol, it is recommended to temper the reagents.

### 2.5. Statistical Analysis

GraphPad Prism 9 (San Diego, CA, USA) and IBM SPSS statistics software version 27 (IBM Corp., Armonk, NY, USA) were used to analyse the experimental data. The results are expressed as mean ± standard deviation (SD). Pearson’s correlation coefficient was calculated using IBM SPSS statistics version 27.

## 3. Results

### 3.1. Participant Characteristics

The venous serum of 123 participants was collected to evaluate the optimized ELISA-based serology protocol performance compared to a commercial ELISA kit. Baseline characteristics of participants in the study are shown in Table 1**.** Between the participants, 68% were men and 32% were women. The median (interquartile range: IQR) age was 64 (56–72) years, being lower in women (median: 58 years) than in men (median: 66 years) (Table 1).

All participants were analysed by a nasopharyngeal swab reverse-transcription quantitative real-time polymerase chain reaction (rRT-PCR) to detect SARS-CoV-2. Ninety-eight of them were hospitalized due to COVID-19-related symptoms and obtained a positive result for SARS-CoV-2 in an rRT-PCR test, while twenty-five patients had no symptoms of COVID-19 and were SARS-CoV-2 negative in rRT-PCR.

### 3.2. Analytical Characteristics of the Optimized ELISA-Based Serology Assay

We developed an indirect ELISA protocol to measure IgG anti-SARS-CoV-2 levels, using the receptor-binding domain (RBD) of the recombinant spike S1 protein of the coronavirus to cover the 96-well plate, as detailed in Materials and Methods. Briefly, once the wells were covered with the RBD of the S1 protein, increasing concentrations of a standard human anti-S1 IgG (in the optimizing phase) or human sera (in the testing phase) were added, incubated for 30 min, and washed. The secondary antibody was an anti-human IgG conjugated with horseradish peroxidase (HRP). The assay was developed using TMB (3,3′,5,5′-Tetramethylbenzidine) as a substrate for HRP, and the optical density (O.D.) was read at 450 nm. A schematic procedure is represented in Figure 1. 

**Figure 2:**
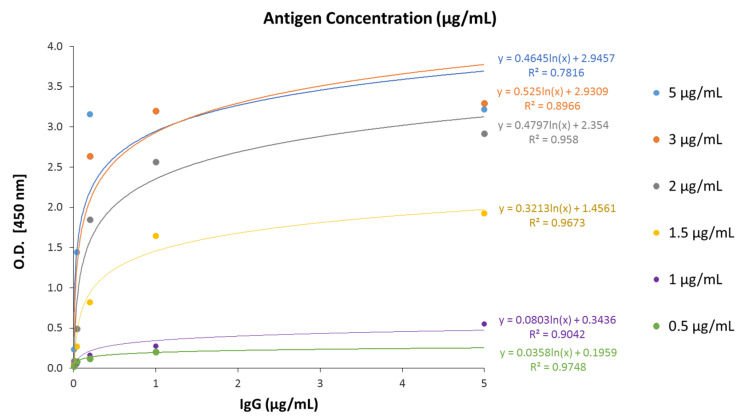
**Calibration curves of the optimized ELISA-based serological assay using different antigen concentrations.** Six concentrations (0.5 µg/mL, 1 µg/mL, 1.5 µg/mL, 2 µg/mL, 3 µg/mL, and 5 µg/mL) of the receptor-binding domain (RBD) of the spike protein of SARS-CoV-2 were used to coat the plates. Then, in each plate, a calibration curve with human IgG (0–5 µg/mL) was determined. The equation for the adjustment to a logarithmic curve, along with the corresponding coefficient of determination (R^2^, R squared), appears on the right.

Firstly, we assayed different concentrations of recombinant RBD of the spike protein, ranging from 0.5 µg/mL to 5 µg/mL, to cover the plate. In all of the RBD concentrations, we determined the calibration curve for human IgG (0–5 µg/mL). As shown in Figure 2, all of the assayed concentrations produced a hyperbolic calibration curve. At 3 µg/mL of RBD, the assay was saturated since either coating the plate with 3 µg/mL or with 5 µg/mL of RBD a similar calibration curve was obtained (Figure 2). Besides, since the saturation occurs from near the lower IgG concentration, the adjustment of datapoints to a logarithmic curve is reduced, showing R squared values lower than 0.9. Then, we choose 2 µg/mL antigens to cover the plate since that condition produced the highest signal before saturation.

**Figure 3:**
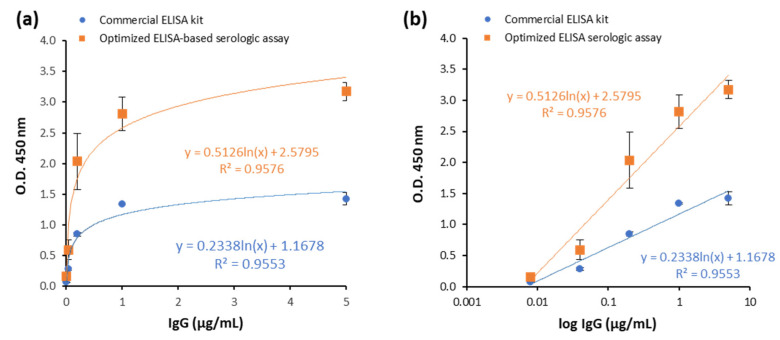
**Comparison of the calibration curves of the commercial ELISA kit and the optimized ELISA-based serological assay.** (**a**) Hyperbolic representation. (**b**) Linear representation using a logarithmic scale in the X axis. Datapoints of the commercial kit are indicated as orange squares, while those of the optimized assay are indicated with blue circles.

Then, we compared the calibration curve of our assay with the calibration curve of a commercial ELISA kit for human anti-S1 IgG determination (see Materials and Methods for further details). Both standard curves were hyperbolic (Figure 3a). However, the signal window of the optimized assay was higher than that of the commercial kit, since in our assay O.D. reads at 450 nm reached values around 3.0, while in the commercial kit, reads barely exceeded values of 1.0–1.5. Moreover, in the commercial kit, curve saturation was reached at 1 µg/mL IgG but not in the optimized ELISA-based assay, where the curve was still rising and not stabilized. Representation in a logarithmic scale clearly shows that the analytical sensitivity of the method (i.e., line slope) of the optimized ELISA-based serologic assay is higher than the analytical sensitivity of the commercial kit (Figure 3b). For assay validation, the limit of detection (LOD), defined as the lowest concentration that can be reliably distinguished from the blank, was calculated by measuring four replicates of a blank (PBS) and calculating the concentration based on a signal of two standard deviations (SDs) above the mean of the blank [28]. The LOD was determined to be 0.0075 µg/mL for the optimized assay, which was comparable to the commercial ELISA kit (0.0079 µg/mL) and is within the established acceptance criteria. In order to calculate the precision of the assay, two different samples were measured by duplicate on three different days. Precision, expressed as the coefficient of variation (%CV), was 14%, comparable to that of the commercial kit (15%).

Next, we tested different human sample dilutions to optimize the serological assay. To that end, eight venous serum samples were chosen arbitrarily, diluted at 1:50, 1:200, 1:500, 1:800, and 1:1000 to be assayed in duplicate with the optimized ELISA-based serological assay. Results are shown in Table 2, in comparison to the values obtained with the commercial ELISA kit with the sample diluted to 1:100, since this was the manufacturer’s recommended dilution. Overall, the O.D. values were always higher in the optimized serology than in the commercial one, even when comparing the measurements of each sample at a dilution of 1:1000 with the optimized assay versus 1:100 with the commercial kit. This fact is explained by the higher analytical sensitivity of the assay presented in this paper and constitutes an important advantage, permitting to test antibody levels even with a scarce volume of human samples. 

On the other hand, the sample dilution that produced better results in the optimized serology was 1:1000. This dilution enables to visualize differences between samples and the values correlated with that obtained with the commercial kit, although, as expected, optical density values were higher in the optimized ELISA-based assay. Samples 1, 3, and 5 were saturated even at the maximum dilution, by approaching the maximum value of the photometric range (i.e., 3.500). In line with this observation, these samples reflect the magnitude of the different sensitive capacities of both methods, through the comparison of raw O.D. data when following the manufacturer’s instructions in the case of the commercial kit and following the protocol presented in this paper in the case of our optimized assay. It should be pointed out that with lower dilutions (1:50, 1:200, 1:500, and 1:800), saturation was produced in most samples (Table 2).

These results indicate that the ELISA-based serological assay was suitable to determine anti-SARS-CoV-2 human IgG levels in serum samples using an antigen concentration of 2 µg/mL (0.2 µg/well) and serum sample dilution of 1:1000.

### 3.3. Assay Performance of the Optimized ELISA Serologic Assay Compared to Commercial ELISA Kit

Once our method was refined, serum samples of 123 participants were tested with the optimized ELISA-based serology assay and with the commercial ELISA kit to validate the assay performance of our method. As shown in Table 3, the diagnostic performance was similar under both protocols, with a difference only in one sample, which was positive with the commercial kit and negative with our serological methodology.

All patients with a positive rRT-PCR were hospitalized with COVID-19-related symptoms. The discrepancy between rRT-PCR and serology data corresponds to the different parameters measured by each methodology. In the case of rRT-PCR, it is the presence of SARS-CoV-2 nucleic acids in the venous serum samples, and then the viral infection, while the serology data determine the presence of anti-RBD S1 SARS-CoV-2 IgG antibodies, so it reflects the immune system response to infection. A positive rRT-PCR and negative serology can occur in the early phases of infection, when antibodies have not been already produced, and a negative rRT-PCR and positive serology can occur after clearing the viral infection when the immune response is still maintained. The commercial ELISA kit was introduced in the experiment for the sake of comparison, although differences between both protocols could be due to diagnostic sensitivity or specificity issues. In any case, either the optimized ELISA-based serology assay or the commercial kit showed quite similar diagnostic performances, differing in 1 of 123 samples (less than 1%).

In addition, we tested whether heat inactivation of venous serum samples at 56 °C for 30 min had any effect on IgG determination. This treatment allows the inactivation of the virus and might be useful in establishing biosafety measures in laboratories. We found similar O.D. values before and after heat inactivation of serum at 56 °C for 30 min, measured under the optimized ELISA-based serological assay (Supplementary Table S1) and therefore this method could be used to reduce the risk of transmission. 

Then, we compared the economic cost of both procedures. It is worth noting that the optimized ELISA-based serological assay, in addition to its higher analytical sensitivity and similar accuracy, was notably cheaper than the commercial kit. The price of a p96 plate was around EUR 1092 for the IgG commercial ELISA kit (depending on national VAT or specific agreements or discounts with the brand), while the price using our optimized ELISA serology was EUR 23.74. Hence, the cost of the optimized ELISA-based protocol was EUR 1.48 per sample (considering the wells needed for controls and the standard curve), sixth-tenfold lower than the commercial kit (i.e., EUR 68.32 per sample). In other words, our optimized serology allows the analysis of 16 samples for the price of each one in the commercial kit, which is a considerable advantage in situations of constrained public spending or economic restrictions. Besides, the fact that our method utilizes samples at a dilution of 1:1000 due to its higher analytical sensitivity offers an additional benefit when the sample size is scarce or the availability of samples is restricted. Taking into account that the method described here provides a well-known step-by-step protocol and reagents can be obtained in most research laboratories, this approach can facilitate the surveillance of future pandemics even in case of reagent shortages due to breaking stocks or transports disabilities. 

Finally, we tested the suitability of the optimized ELISA-based assay to detect anti-SARS-CoV-2 IgGs in self-collected capillary blood samples. For that purpose, we choose 40 volunteers who self-collected a sample of capillary blood as indicated in Materials and Methods. As blood contains not only liquid but also a cellular fraction, and then analytes are usually more diluted in blood than in serum, we decided to perform the optimized ELISA-based serology using a capillary blood dilution of 1:200, instead of 1:1000. In that case, for the sake of comparison, capillary serum was eventually diluted to 1:200. Serology was also performed with capillary serum diluted to 1:500 and 1:1000 (data not shown), and dilutions above 1:500 are not recommended for capillary serum. 

**Figure 4:**
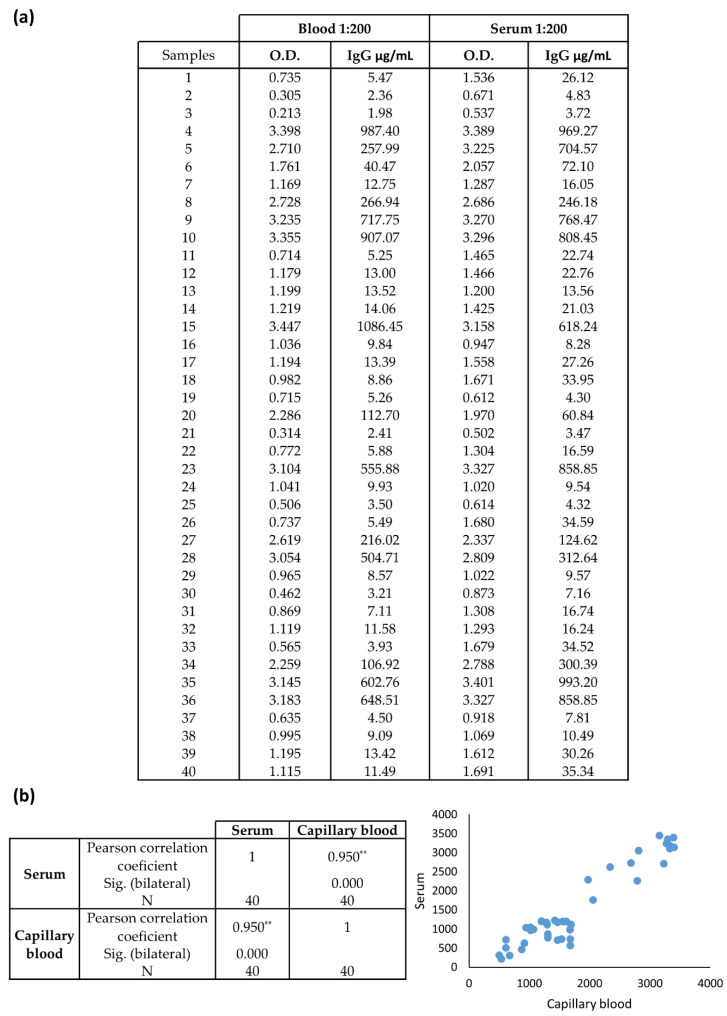
Comparison of determination of IgG in capillary blood and serum samples with the optimized ELISA-based serological assay. (**a**) Values of O.D. at 450 nm and the corresponding IgG concentration (µg/mL) shown in 40 samples. (**b**) The linear relationship between O.D. values of matched serum and blood capillary samples. ** Correlation is statistically significant at level 0.01 (bilateral).

The data obtained with the optimized ELISA-based assay showed that the concentration of IgGs in capillary blood was approximately half of the concentration in serum, by mean (Figure 4a). However, a high level of agreement between capillary serum and their matched capillary blood samples was observed. Correlation analysis indicated a positive correlation between values in serum and blood capillary samples, with a Pearson’s coefficient of 0.95, being statistically significant (Figure 4b). 

To sum up, our results indicate that the ELISA-based serological assay described in this paper is suitable for anti-SARS-CoV-2 IgG determination at a low cost and it can be used in point-of-care settings.

## 4. Discussion

This paper describes the optimization of an ELISA-based serology assay for the detection of IgG antibodies against the RBD of the S1 spike protein of SARS-CoV-2 in human samples. The assay performance was also compared to a commercially available ELISA kit and, in the validation, our protocol was found to be suitable for its intended purpose. Here, we demonstrate the validity of our cheap immunoassay for the measurement of specific IgG in venous serum samples of patients, which allows the safe and economic testing of currently vaccinated people to assess immunity in the population and the efficacy of vaccination. Results of the optimized ELISA-based serology assay showed almost perfect agreement and high correlations of diagnostic sensitivity with results from the commercial ELISA kit, differing in only 1 of 123 samples. Interestingly, the optimized assay had higher analytical sensitivity than the commercial kit and it was used not only in venous serum but also in finger-stick capillary blood and capillary serum samples. 

Our study showed that finger-stick capillary blood can serve as a suitable source of IgG detection, bypassing the need for serum isolation and being suitable for point-of-care application. Venous whole blood assays are limited in scale due to the requirement for a phlebotomist to obtain samples, while capillary blood-based sampling becomes a highly attractive alternative as it can be carried out quickly and safely by patients themselves. It is worth noting that the assay takes tiny amounts of blood that can be obtained easily by finger-stick and is a reliable and feasible alternative to venepuncture for serological assessment of COVID-19. This simple and inexpensive method is suited for the study of SARS-CoV-2 seroprevalence, particularly where there is a lack of trained healthcare staff or geographically dispersed populations. Furthermore, a recent study has reported that when the samples are professionally collected, the categorical agreement between anti-SARS-CoV-2 antibodies obtained from dried blood spots and venous serum only increases by 2% from >98% to 100%, validating this monitoring method for at-home collection [29]. Another benefit of the finger-stick blood test is that it can be adapted for assessing the neutralizing antibody response against wild-type, alpha, beta, gamma, and delta variant RBDs [30]. 

Very recently, several reports have demonstrated the possibility of using capillary blood instead of serum for IgG evaluation [31,32,33]. In fact, it has been described that the storage of capillary blood at room temperature for up to 7 days post-sampling did not affect the result [32], which reaffirms this approach as a viable alternative. Our results are in concordance with those studies and demonstrate an affordable protocol for measuring SARS-CoV-2 antibodies in various resource settings, in particular in those where common supplies needed for the classical methods based on kits are in shortage. In this sense, our method shows an advantage versus more recent publications based on commercial serological tests [34,35]. Moreover, since it is a sensitive and cheap method, it could improve access to serological testing and can be used for massive screening. Therefore, our cheap immunoassay may be useful to provide population-based data on SARS-CoV-2 seropositivity, infection, and immunity generated by the vaccines and, more importantly, the assay may be used by economically less-favoured nations or in settings with supply shortages, as occurred in the first phases of the present pandemic. 

During this pandemic, as in other viral infectious outbreaks causing health concerns, all of the biological samples were considered potentially dangerous while infectiveness was tested. In addition to protective equipment and qualified laboratories, viral inactivation procedures (e.g., chemical or physical) are recommended prior to handling samples in order to increase the safeness of the experimental conditions, reducing personal risks while preserving the specimens’ key features (e.g., RNA for PCR testing or antibodies for serology assays). In this study, we determined the IgG levels in 73 venous serum samples before and after heating them for 30 min at 56 °C. This temperature was chosen because 56 °C is commonly used for the inactivation of enveloped viruses, including coronaviruses, such as SARS [36,37,38,39]. Furthermore, Batéjat et al. recently reported that no infectious SARS-CoV-2 was detected when heating samples at 56 °C within 30 min in culture medium, 20 min in nasopharyngeal samples, and 15 min in sera, using the TCID_50_ method for testing infectivity [40]. Our results show quite similar optical density values before and after heating, measured under the optimized ELISA-based serological assay, indicating that this physical procedure did not affect the levels of antibodies present in the samples. In fact, many samples displayed slightly higher values after heating. In line with this result, another study reported that 12 of 34 human samples with COVID-19 had increased IgG signals after heating, with a median percentage of 24.22%, using a commercial antibody detection kit for SARS-CoV-2 based on fluorescence immunochromatography (AIE/quantum dot-based fluorescence immunochromatographic assay, AFIA) [41]. These authors hypothesized that this augmentation could be due to the formation of IgG aggregates at 56 °C, which increased the immunogenicity. To sum up, the observation of similar values before and after heating indicates that samples can be inactivated without perturbing the nature or quantification of these antibodies.

Within the possible limitations of the assay, we can mention the availability of the recombinant protein used as antigen for coating the plaque. However, the advancements in biotechnology and molecular biology tools can help hamper this putative constriction. Moreover, a higher number of samples would be desirable, although it should be highlighted that the optimized ELISA-based serology test was carried out with more than a hundred human samples, obtained during 1–30 June 2020, barely three months after the lockdown in most of the world due to the outbreak of SARS-CoV-2 infection and still in the most critical months of the pandemic. Lastly, although healthcare professionals are not needed, technical or scientific personnel with certain skills for research are required in order to follow the protocol. On the other hand, the relevance of this study is dual. Firstly, it provides a cheaper ELISA-based serology for the detection of anti-RBD SARS-CoV-2 antibodies, which allows realizing massive screenings and its implementation in point-of-care settings. Secondly, it is a detailed procedure that can be applied in the absence of commercial kits, for example, due to a break of stocks or reagent shortages, while using common reagents in a research laboratory. This information may be useful in the case of future pandemics with novel viruses. 

## Figures and Tables

**Table 1:** Baseline characteristics of participants in the study. Values are numbers (percentages) or median (interquartile range).

Features	Total
Participants (*n*, %) • SARS-CoV-2 PCR + • SARS-CoV-2 PCR −	123 (100%) • 98 (80%) • 25 (20%)
Sex (*n*, %) • Male • Female	123 (100%) • 84 (68%) • 39 (32%)
Median age (IQR) (years) • Male • Female	64 (56–72) • 66 (56–72) • 58 (57–69)

**Table 2:** **Determination of proper human serum dilution, performed in the optimized ELISA-based serology assay.** Optical density values are shown for eight randomly chosen samples assayed in duplicate. Different sample dilutions were measured with the optimized serology assay in comparison with the commercial ELISA kit at the manufacturer’s recommended dilution. Results are the mean ± S.D.

	Commercial ELISA Kit	Optimized ELISA-Based Serological Assay				
Samples	1:100	1:50	1:200	1:500	1:800	1:1000
1	1.569 ± 0.047	3.324 ± 0.037	3.330 ± 0.006	3.317 ± 0.006	3.281 ± 0.004	3.298 ± 0.025
2	0.884 ± 0.009	3.396 ± 0.061	3.365 ± 0.006	3.107 ± 0.030	3.165 ± 0.058	2.897 ± 0.052
3	1.604 ± 0.003	3.357 ± 0.001	3.447 ± 0.002	3.440 ± 0.047	3.321 ± 0.013	3.379 ± 0.028
4	0.178 ± 0.005	3.194 ± 0.025	3.149 ± 0.054	1.793 ± 0.031	1.016 ± 0.095	0.809 ± 0.018
5	0.921 ± 0.023	3.471 ± 0.026	3.450 ± 0.006	3.404 ± 0.018	3.302 ± 0.063	3.332 ± 0.008
6	0.154 ± 0.000	3.178 ±0.006	3.228 ± 0.013	1.778 ± 0.028	1.115 ± 0.122	0.791 ± 0.014
7	0.051 ± 0.001	1.958 ± 0.018	0.895 ± 0.032	0.357 ± 0.018	0.280 ± 0.011	0.269 ± 0.013
8	0.027 ± 0.002	1.322 ± 0.066	0.903 ± 0.041	0.403 ± 0.004	0.306 ± 0.011	0.306 ± 0.040

**Table 3:** Assay performance determination of the optimized ELISA-based serology and the commercial ELISA kit in 123 venous serum human samples.

Participants	rRT-PCR	Optimized ELISA-Based SEROLOGY Assay	IgG Commercial ELISA Kit
123	Positive 98 (80%)	Positive: 74 (76%) Negative: 24 (24%)	Positive: 75 (77%) Negative: 23 (23%)
	Negative 25 (20%)	Positive: 3 (12%) Negative: 22 (88%)	Positive: 3 (12%) Negative: 22 (88%)
	Total	Positive 77 Negative 46	Positive 78 Negative 45

## Data Availability

The data used to support the findings of this study are deposited in https://data.mendeley.com/datasets/DOI: 10.17632/k6p2b7nmy2.

## Supplementary Materials

The following supporting information can be downloaded at: https://www.mdpi.com/article/10.3390/biomedicines10112897/s1, Table S1: O.D. of venous serum human samples before and after heating at 56 °C for 30 min, determined with the optimized ELISA-based serological assay.
