# Supplementary material for: A Highly Sensitive Immunoassay for Determination of Immune Response to SARS-CoV-2 in Capillary Blood Samples
Source: Biomedicines. 2022 Nov 11;10(11):2897. doi: 10.3390/biomedicines10112897 (PMC9687217; doi:10.3390/biomedicines10112897)
Supplement: Supplementary file 1 [file biomedicines-10-02897-s001.zip › biomedicines-1974238-supplementary.pdf]

Table S1. O. D. of venous serum human samples before and after heating at 56 °C for 30 min, determined with the optimized ELISA-based serological assay.

Samples (1-36)	O. D. (450 nm) Before heating	O. D. (450 nm) After heating
1	3.226	3.053
2	1.914	1.532
3	2.107	1.996
4	3.239	2.886
5	0.441	0.379
6	2.676	2.447
7	0.705	0.499
8	0.969	0.616
9	0.118	0.133
10	1.165	1.046
11	0.038	0.122
12	2.444	2.526
13	3.084	2.921
14	2.949	2.653
15	2.383	2.512
16	3.127	2.753
17	0.449	0.687
18	1.433	1.401
19	2.177	2.253
20	0.428	0.383
21	2.750	2.696
22	0.882	0.985
23	3.291	3.330
24	0.444	0.631
25	3.184	3.111
26	0.937	0.953
27	3.131	2.938
28	3.310	3.157
29	0.711	0.855
30	1.843	2.231
31	0.472	0.823
32	3.279	3.075
33	2.517	2.520
34	1.461	1.493
35	2.859	2.788
36	2.361	2.606
37	2.765	2.940

Samples (37-73)	O. D. (450 nm) Before heating	O. D. (450 nm) After heating
38	2.638	2.564
39	1.388	1.520
40	1.261	1.274
41	3.355	3.331
42	0.161	0.329
43	1.718	2.655
44	3.330	3.298
45	2.943	2.676
46	1.351	1.707
47	3.208	3.200
48	0.109	0.227
49	3.171	3.330
50	3.381	3.339
51	0.337	0.619
52	3.373	3.339
53	0.358	0.546
54	1.177	1.568
55	0.904	1.919
56	3.167	3.274
57	3.197	3.323
58	1.676	2.781
59	0.001	0.037
60	1.772	2.407
61	3.327	3.244
62	3.078	3.245
63	3.375	3.333
64	3.324	3.342
65	2.689	3.179
66	1.590	2.484
67	0.576	0.756
68	2.596	2.944
69	0.567	0.801
70	0.756	1.260
71	2.353	3.096
72	3.295	3.235
73	3.349	3.316
